# Developmental dyslexia: predicting individual risk

**DOI:** 10.1111/jcpp.12412

**Published:** 2015-04-02

**Authors:** Paul A Thompson, Charles Hulme, Hannah M Nash, Debbie Gooch, Emma Hayiou-Thomas, Margaret J Snowling

**Affiliations:** 1 University of OxfordOxford, UK; 2 University College LondonLondon, UK; 3 University of LeedsLeeds, UK; 4 Royal HollowayLondon, UK; 5 University of YorkYork, UK

**Keywords:** Familial (family) risk, dyslexia, reading disability, language skills, executive motor, early identification

## Abstract

**Background:**

Causal theories of dyslexia suggest that it is a heritable disorder, which is the outcome of multiple risk factors. However, whether early screening for dyslexia is viable is not yet known.

**Methods:**

The study followed children at high risk of dyslexia from preschool through the early primary years assessing them from age 3 years and 6 months (T1) at approximately annual intervals on tasks tapping cognitive, language, and executive-motor skills. The children were recruited to three groups: children at family risk of dyslexia, children with concerns regarding speech, and language development at 3;06 years and controls considered to be typically developing. At 8 years, children were classified as ‘dyslexic’ or not. Logistic regression models were used to predict the individual risk of dyslexia and to investigate how risk factors accumulate to predict poor literacy outcomes.

**Results:**

Family-risk status was a stronger predictor of dyslexia at 8 years than low language in preschool. Additional predictors in the preschool years include letter knowledge, phonological awareness, rapid automatized naming, and executive skills. At the time of school entry, language skills become significant predictors, and motor skills add a small but significant increase to the prediction probability. We present classification accuracy using different probability cutoffs for logistic regression models and ROC curves to highlight the accumulation of risk factors at the individual level.

**Conclusions:**

Dyslexia is the outcome of multiple risk factors and children with language difficulties at school entry are at high risk. Family history of dyslexia is a predictor of literacy outcome from the preschool years. However, screening does not reach an acceptable clinical level until close to school entry when letter knowledge, phonological awareness, and RAN, rather than family risk, together provide good sensitivity and specificity as a screening battery.

## Introduction

Dyslexia is a specific learning disorder which runs in families; the consensus view for many years has been that it is the behavioral outcome of an underlying phonological deficit (Peterson & Pennington, 2012; Snowling & Hulme, 2012; Vellutino, Fletcher, Snowling, & Scanlon, 2004; for reviews). In DSM-5 (American Psychiatric Association, 2013), dyslexia is classified with other neurodevelopmental disorders which have early onset. A major issue therefore is whether the early identification of dyslexia is practicable, before a downward spiral of poor reading, poor attainment, and career prospects is established. Longitudinal studies following children from preschool through formal reading instruction speak directly to this issue.

Studies of the variations in reading skills in unselected samples of children typically begin when children are in the year prior to school entry. Those which have been conducted in alphabetic languages converge on the view that there are three predictors of individual differences in children’s decoding, word recognition skills, and reading fluency: letter knowledge, phoneme awareness, and rapid automatized naming (RAN) (e.g., Caravolas et al., 2012). Less attention has been paid to the precursors of these crucial foundations (c.f. Carroll, Snowling, Hulme, & Stevenson, 2003). However, two large scale studies following children from age three reported that early language skills predicted individual differences in phonological awareness and letter knowledge which, in turn, predicted reading (NICHD, 2005; Storch & Whitehurst, 2002); this study also reported a direct effect of language on later word decoding. It follows that oral language difficulties beyond specific phonological processes may be additional risk factors for dyslexia. Consistent with this, children with specific language impairment are at high risk of dyslexia (Bishop & Snowling, 2004).

Longitudinal studies following children at family risk of dyslexia because they have an affected relative are also relevant to early identification. While the cutoff for identifying dyslexia varies between studies, the prevalence of dyslexia is higher in those at familial risk, rendering it a significant risk factor, regardless of language and school system (Snowling & Melby-Lervag, unpublished data[Bibr b34]). Furthermore, the consensus is that the early signs of dyslexia include delays in speech and language development with phonological memory (nonword repetition) and expressive language (naming) skills being particularly affected (e.g., Carroll, Mundy, & Cunningham, 2014). Consistent with earlier work showing that RAN, letter name knowledge, and phonological awareness are core predictors of dyslexia (Catts, Fey, Zhang, & Tomblin, 2001; Pennington & Lefly, 2001), these studies highlight the slow development of language, phonological awareness, and decoding-related skills, including poor rapid naming, in children at family risk of dyslexia (Torppa, Lyytinen, Erskine, Eklund, & Lyytinen, 2010).

However, knowledge of the precursors of dyslexia does not directly address the issue of early identification. It is important to assess not only the probability that a set of measures will identify positive cases of dyslexia (sensitivity) but also the probability that it will avoid false positives (specificity). Pennington and Lefly (2001) reported that the best-fitting model of literacy outcome in an English-speaking family risk sample included letter name knowledge, IQ, speech perception, phonological awareness, verbal STM, and rapid naming (RAN) measured at 5 years and ‘risk’ according to a reading history questionnaire. The model yielded 69% sensitivity and 76% specificity. Similarly, Elbro et al., (1998) reported sensitivity of 78% and specificity of 79% in a Danish family risk study. The predictors in this model were letter naming, initial-phoneme deletion, phoneme identification, pronunciation accuracy, and distinctness of phonological representations measured in kindergarten at age 6.

In a more comprehensive analysis, Puolakanaho et al. (2007) investigated the predictors of dyslexia in Finnish-speaking children using measures of letter name knowledge, phonological awareness, rapid naming, short-term memory, expressive vocabulary, and nonword repetition (not all available from all time points). Age-specific logistic regression models revealed that familial risk status and letter name knowledge measured at ages 3½, 4½, and 5½ years were significant predictors of dyslexia in Grade 2; phonological awareness and rapid naming were additional predictors at ages 4½ and 5½, respectively. Importantly, prediction was best for models containing several measures and it was notable that different ‘risk’ factors interacted to determine outcome.

This study aimed to extend the work of Puolakanaho et al., to an English-speaking sample of children. A novel feature was that the high-risk sample here included children with preschool language impairment as well as children at risk of dyslexia by virtue of having a first degree affected relative, and some children were both language impaired and at family risk. The study was cast within the multiple risk framework of Pennington and colleagues (e.g., Pennington, 2006; Pennington et al., 2012): since executive and motor deficits are common among children with language difficulties (Gooch, Hulme, Nash, & Snowling, 2014; Henry, Messer, & Nash, 2012) and deficits in self-regulation, attention control, and fine motor coordination are likely to affect the learning to read and spell (e.g., Spira & Fischel, 2005), we included measures of executive function and motor skills in addition to known precursors of dyslexia (letter knowledge, phoneme awareness and RAN). Our aim was to identify a set of predictors of dyslexia (defined by poor word reading and spelling) and to estimate the risk to an individual at different developmental stages.

## Methods

### Participants

Families were recruited to the study via advertisements in newspapers, nurseries, and the webpages of support agencies for children with reading and language difficulties and speech and language therapy services to three groups: children from families with a history of dyslexia/reading problems, children for whom there were concerns regarding speech and language development, and controls considered to be typically developing. All children spoke English as their first language and the large majority were white British. The full range of socioeconomic status was represented in the sample, although all variables showed a negative skew, indicating relatively high average SES. Parental educational level and occupational status were significantly higher in the control than the risk groups.

Following recruitment, children were classified using a two-stage process, determining whether they were at family risk (FR) of dyslexia and then using diagnostic criteria to ascertain whether they had language impairment (SLI) (see Nash, Hulme, Gooch, & Snowling, 2013 for details). This procedure yielded four groups: FR-only (*N* = 86), SLI (*N* = 36), FR-SLI (*N* = 37), control (*N* = 71); 15 children with concerns regarding speech and language development did not meet inclusionary criteria for SLI but for the purposes of this study, were included in the sample. The children were assessed at approximately yearly intervals between T1 (∼age 3½) and T5 (∼age 8). There was a small amount of attrition between time points (*N* = 21) and 15 children entered the project at the second time point; see the online supplementary material (Appendix S1 for details of participant flow. The total sample of 260 children was included in the present analyses.

Ethical permission was granted by the University of York, Department of Psychology’s Ethics Committee and the NHS Research Ethics Committee. Parents provided informed consent for their child to be involved.

### Tests and procedures

Each child was administered a comprehensive battery of cognitive, language, and literacy tests at each time point. Here, we only report details of the measures which are used in the present analyses.

#### Nonverbal ability

Nonverbal IQ was measured using two subtests from the Wechsler Preschool and Primary Scale of Intelligence (WPPSI-III; Wechsler, 2004); Block Design (α* *= .86) and Object Assembly (α* *= .85). The mean of *z*-standardized scores for the two subtests provided a composite nonverbal IQ score.

#### Language measures

*Basic concepts (CELF-Preschool 2 UK;* Wiig, Secord, & Semel, 2006*) (T1)*. The child hears a sentence (e.g., point to the one that is long) and has to select from a choice of three, the picture that demonstrates the concept (α* *= .82).

*Expressive vocabulary (CELF-Preschool 2 UK; Wiig* et al.*,* 2006*) (T1, T3, T4)*. The child is asked to name objects (e.g., carrot, telescope) or to describe what a person in the picture is doing (e.g., riding a bike) (α* *= .78–.82).

*Receptive vocabulary (Receptive one word picture vocabulary test (ROWPVT) Brownell,* 2000*) (T2, T4)*. The child hears a word and selects the corresponding picture from four choices (α = .95).

*Sentence structure (CELF-Preschool 2 UK; Wiig* et al.*,* 2006*) (T1, T2, T3)*.The child hears a sentence (e.g., the bear is in the wagon) and selects from four, the corresponding picture (α = .78–.83).

*Test of reception of grammar (TROG-2; Bishop,* 2003) (T4) The child hears a sentence and selects the corresponding pictures from four comprising lexical and grammatical foils (α = .88).

*Sentence repetition* (T2, T3, T4) This measure, designed for this study, requires the child to repeat 20 sentences: 10 (5 long/5 short) containing transitive verbs and 10 (5 long/5 short) containing ditransitive verbs (α = .78).

*Word structure (CELF-Preschool 2 UK; Wiig* et al.*,* 2006*) (T4)* The child is shown two pictures, the examiner describes the first one and the child has to describe the second (e.g., irregular plural ‘here is one mouse, here are two …’) (α =.78).

#### Phonological measures

*Word and nonword repetition (Early Repetition Battery; Seeff-Gabriel, Chiat, & Roy,* 2008*) (T1)*. The child repeats 18 words (6 one-syllable, 6 two-syllable, and 6 three-syllable) and 18 nonwords (6 of each length) (α = .89).

*Alliteration matching (Carroll* et al., 2003*) (T2)* The child identifies which of two words starts with the same sound as a target word for 10 items (e.g., pot: duck or peach) (α = .67).

*Phoneme isolation of initial and final sounds* (*YARC; Hulme* et al.*, 2009) (T2, T3)* The child repeats a nonword and then has to say its first sound. There are nine monosyllabic test items, 4 CVC and 4 CCVC. Following the initial isolation task, the child says the last sound in the nonword (4 CVC and 4 CVCC items). For both tasks, testing is discontinued after four incorrect responses. (α = .88).

*Phoneme deletion* (*YARC; Hulme* et al.*, 2009) (T3, T4)* The child hears a word, repeats it, and then says it dropping a specified phoneme (e.g., ‘without the/b/’) (12 items) (α = .93).

#### Rapid automatized naming (RAN)

*RAN colors (T2)* Children first name each of the five stimuli (squares colored brown, blue, black, red, and green) to check that they know them. Following this, they are presented with an 8 × 5 array of stimuli and asked to name each of the stimuli (moving from left to right) as quickly as possible. The time taken to name all 40 stimuli and the number of errors made are recorded. RAN rate is calculated (number correct (max 40)/time(s)).

*RAN objects (T2,T4)* The test was the same as for RAN colors, but the items were pictures of a dog, eye, key, lion, and table (reliabilities based on time for two halves were *r* = .71).

*RAN digits (T4)* The test was the same as for RAN colors and objects, but the items were digits: 2, 1, 5, 8, 4 (reliability = .75).

#### Executive skills

*Go/No-Go task (T1)* In this adaptation of the Bear-Dragon Go/No-Go task (Reed, Pien, & Rothbart, 1984, the child follows verbal instructions (e.g., ‘thumbs up’) given by one puppet (a bird) while responding to commands given by another (a dog). An efficiency score (Hits: number of responses to the dog (max 8)/total number of responses (responses to dog + bird (max 16)) is calculated; a high score reflects better behavioral inhibition.

*Heads-Toes-Knees-and Shoulders (HTKS) task* (Burrage et al., 2008; Cameron Ponitz et al., 2008) *(T1, T2, T3)* The child hears a command and performs the opposite (e.g., touch toes if asked to touch head and vice versa). If the child completes 5/10 trials successfully inhibit on 5/10 trials, they are asked to touch shoulders, knees etc. Each correct response – 2 points, self-corrected responses – 1 point (max score = 40). Stability between T1 and T2; *r *=* *.51.

*Visual Search task (Apples Task;* Breckenridge, 2008*). (T1, T2, T3, T4)* The child is given 1 min to search an array and point to red apples, ignoring red strawberries, and white apples. A visual search efficiency score ((Hits: total targets correctly identified – commission errors)/60 s) is calculated; a high score reflects better selective attention. Stability between T1 and T2; *r *=* *.53.

*Block Recall (Working Memory Test Battery for Children, Pickering & Gathercole,* 2001*) (T2, T3, T4*). To assess visuo-spatial working memory, the child watches the examiner tap a sequence of blocks and then recalls the sequence by tapping the blocks in the same order. Test–retest reliability = 0.63.

*Auditory Continuous Performance Test (ACPT) (T2, T3)*. In this task (after Kerns & Rondeau, 1998), the child sees the image of a farm and hears four animal sounds (cow, duck, frog, dog). The child is asked to press a button when the dog barks. To make the task suitable for younger children, the target distractor ratio was reduced and since it was conducted in the auditory domain, the length of the task was limited to 5 min. A sustained attention efficiency score ((Hits: total targets correctly identified – commission errors)/120 trials) was calculated.

#### Motor skills

*Movement Assessment Battery for Children-2 (Henderson, Sudgen, & Barnett,* 2007) (*T1,T2,T3,T4)*. The child completed three tests of fine motor skill: posting coins, bead threading and bicycle trails (α = .86–.91). At T1, they also copied three shapes: cross, circle, and square, scored out of 3.

#### Literacy measures

*Letter Knowledge (YARC; Hulme* et al.*, 2009)(T1, T2, T3, T4)* The child is shown 32 single letters and digraphs and asked to give the sounds (at T1 only 12 letters). If a letter name is given, the child is prompted to provide the sound (α =.95).

*Word Reading (SWRT; Foster,* 2007*) (T5)* The child reads aloud 60 words of increasing difficulty. Testing is discontinued after five consecutive errors or refusals. (α = .98).

*Spelling words (WIAT; Wechsler, 2005)(T5)* The child spells a set of words increasing in difficulty. Testing is discontinued after 10 consecutive errors. (α = .93).

## Results

### Data screening and preparation

The predictors used were composite variables with the exception of letter knowledge which was a manifest variable (standardized). At each time point, the composite measures included similar constructs, each standardized and then averaged, but measures were not identical across time. The language composite at each time point consisted of measures of vocabulary and grammar. The phonology composite consisted of measures of repetition at T1 (phoneme awareness was not measurable) and thereafter measures of phoneme awareness. The RAN composite comprised colors and objects at T2 and RAN objects and digits at T4. The executive function construct was based on different measures to allow for developmental changes: T1 – selective attention, inhibition, and self-regulation tasks; T2 and T3 – selective attention, sustained attention, self-regulation, and visuo-spatial memory; T4 – selective attention and visuo-spatial memory. The observed variables in the motor skills composite (copying shapes, coin, beads, bike trails) were skewed and had some outliers at every time point. The outliers were relocated to the tails of the distributions prior to analysis. The composite measure was reversed for interpretation purposes. With the exception of letter knowledge (T3, T4) which was at ceiling and sentence repetition (T2) which showed floor effects, all other measures were well distributed. Summary statistics for all variables are shown in the online supplementary material (Table S1).

A composite literacy measure from T5, the average of the standard scores for word reading and spelling, was used to classify children as dyslexic at 8 years (T5). A cutoff criterion for dyslexia was defined as falling 1.5 *SD*s below the mean standard score of the control group on the T5 literacy composite measure (*M* = 106.88; *SD* = 11.68); this was a standard score of 88 relative to population norms. In the models which follow, complete cases were used, so no missing data were imputed; there were 47 cases classified as dyslexic (13F/34M) and 172 defined as nondyslexic (79F/93M).

### Modelling approach

Using a four stage procedure we tested: (a) a model with two predictors: familial risk of dyslexia and language skills at the ages of 3½, 4½, 5½, and 6–7 years; (b) a model substituting core measures of letter knowledge, phonological awareness and RAN (as available at each time) for the language composite. A further model including language together with these core measures provided a poor fit to the data and hence was not considered further (language was not significant at any time once these ‘core’ measures were included); (c) a model with additional measures of executive and motor skills assessing whether features of comorbidity increase the probability of a child developing dyslexia over core measures at each time. Finally, (d) best-fitting models were used in ROC analyses to investigate the accuracy with which dyslexia could be predicted, and to plot probability curves elucidating individual risk of dyslexia.

Prior to building the logistic regression models, we investigated group-level (dyslexic vs. nondyslexic) differences to aid model specification. Table1 shows the performance of the sample across the candidate predictors, classified according to dyslexia outcome. A series of *t*-tests with corrections for multiple tests (accepting statistical significance at *p *<* *.008) were performed to investigate mean group differences.

**Table 1:** Mean difference between dyslexia and nondyslexia across the predictors

Variables	Time	*N*	Mean (*SD*)		*t*	Cohen’s *d*
			Nondyslexia	Dyslexia		
Girls/Boys	1	219	79/93	13/34	–	–
IQ	1	202	.07 (.84)	−.06 (.82)	.91	.16
Performance IQ age 3.5	1	219	109.36 (15.20)	103.64 (13.19)	−5.71	.42
Performance IQ age 8	5	219	105.39 (14.67)	95.47 (14.22)	−9.92*	.69
Mother’s education	1	219	4.16 (1.48)	3.60 (1.56)	−2.30	.37
Language	1	199	.15 (.80)	−.15 (.83)	2.04	.37
	2	199	.14 (.78)	−.14 (.79)	1.94	.36
	3	215	.16 (.78)	−.41 (.77)	4.32**	.74
	4	218	.21 (.70)	−.56 (.84)	6.32*	1.00
Letter sound knowledge	1	206	.14 (1.06)	−.40 (.63)	3.24*	.62
	2	217	.14 (.94)	−.40 (1.05)	3.40*	.54
	3	219	.28 (.52)	−.92 (1.50)	8.79**	1.07
	4	218	.27 (.38)	−.77 (1.52)	8.16*	.94
Phonology	1	192	.18 (.94)	−.18 (.90)	2.23	.39
	2	172	.24 (.86)	−.44 (.72)	3.83*	.86
	3	219	.24 (.72)	−.77 (.92)	8.04**	1.22
	4	219	.30 (82)	−.97 (.89)	9.26*	1.48
RAN	2	177	.14 (.92)	−.48 (.60)	3.33*	.80
	3	215	.18 (.96)	−.49 (.73)	4.30*	.79
	4	217	.20 (.82)	−.73 (.80)	6.85*	1.15
Executive function	1	139	.17 (.68)	−.14 (.54)	2.36	.50
	2	189	.15 (.68)	−.23 (.67)	3.14*	.56
	3	207	.12 (.60)	−.33 (.79)	4.05**	.64
	4	218	.15 (.73)	−.35 (.94)	3.91*	.59
Motor sills	1	181	−.07 (.45)	−.07 (.50)	0.04	0
	2	215	−.12 (.68)	.26 (.96)	−3.08*	−.46
	3	217	−.10 (.67)	.32 (.94)	−3.42**	−.51
	4	219	−.07 (.71)	.20 (1.02)	−2.126	−.31

All variables used in composites have been z-scored.

* *p *<* *.008;

** *p *<* *.01.

The groups differed significantly in nonverbal IQ at T5 (*d *=* *.69), but not at T1 (*d *=* *.16), therefore, this was not entered as a predictor in the models. At T1, there was only one significant group difference in decoding-related skills, namely letter sound knowledge (*d *=* *.62); the effect sizes for differences in phonology, and executive function were small to medium (*d *=* *.4–.5). At T1 and T2, the effect size for language was small to medium (*d *=* *.36), but it increased at T3 and T4 (*d*s = .74–1). At T2, T3, and T4, groups differed significantly on core predictor measures (letter sound knowledge, phonology, and RAN) (*d*s = .54–1.48) with effect sizes for executive function and motor skills ranging from small to large (*d*s = −.31 to .64).

### Predicting dyslexia using language and familial risk

First, to provide a coarse screening algorithm suitable for clinicians to use for determining risk of dyslexia, we tested a model with only family risk and language composite measures (Model 1). In the models, each variable is entered in separate blocks.

**Figure 1:**
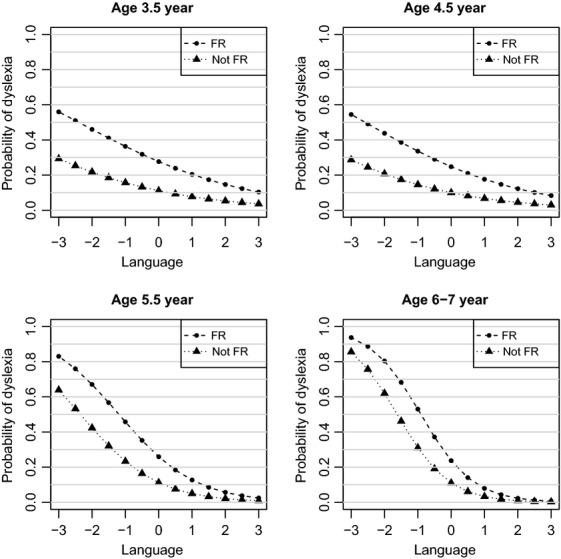
Age-specific probability curves showing the change in risk of dyslexia by language and family risk status
*Notes*: Curves represent changes in probability of identifying dyslexia (RD) at different levels of language (*x-axis*). Values represent high (+3) to low (−3) levels of performance. The curves represent children at family-risk (FR) of dyslexia or not at family risk.

Family risk status was predictive of dyslexia at every time point. Figure1 shows a marked difference between individuals with family risk and those without, the curve for the FR group being higher at every time point. The Nagelkerke *R*^2^ value represents the percentage of total variation in the outcome explained by the model. We found that as the age-specific models approached the outcome age, the *R*^2^ value increased (T1 = 20.3%; T2 = 32.2%; T3 = 38.6%; and T4 = 51.0%).

Table2 (Model 1) shows our initial finding was confirmed – language was not a significant predictor at T1 or T2 when family risk was in the model, but it was a significant predictor at later time points. Figure1 shows the probability curves are flatter at T1 and T2 suggesting that poor language ability in the preschool years is only a weak predictor of dyslexia. In contrast, at T3 and T4, a more exaggerated sigmoidal shape is seen in the probability curve indicating a significant influence of language ability as a risk factor for dyslexia around the time of school entry.

**Table 2:** Best fitting age-specific logistic regression models for prediction of dyslexia at 8 years (T5) from family risk status and language skills (Model 1); family risk, letter knowledge, phoneme awareness, and RAN (Model 2) and with additional predictors (Model 3)

	Variables	Model 1		Model 2		Model 3	
		B (*SE*)	Odds ratio	B (*SE*)	Odds ratio	B (*SE*)	Odds ratio
Time 1 (3.5 years)	Group FR	1.12 (.40)*	3.08	1.34 (.56) *	3.81	–	–
	Language	−0.40 (.22)	0.67	n/a	n/a		
	Letter knowledge	n/a	n/a	−.68 (.34)*	0.51	–	–
	Constant	−2.08 (.34)*	0.12	−2.23 (.49)*	0.11	–	–
Time 2 (4.5 years)	Group FR	1.09 (.42)*	2.99	1.266 (.56)*	3.548	1.19 (.62)**	3.3
	Language	−0.43 (.25)	0.65	n/a	n/a	n/a	n/a
	Letter knowledge	n/a	n/a	.630 (.34)	1.878	1.06 (.40)*	2.88
	Phonology	n/a	n/a	−1.228 (.36)*	0.293	−2.03 (.49)*	0.13
	RAN	n/a	n/a	−.581 (.34)	0.56	−1.69 (.53)*	0.18
	EF	n/a	n/a	n/a	n/a	−1.66 (.66)*	0.19
	Constant	−2.20 (.35)*	0.11	−2.877 (.52)*	0.06	−3.34 (.64)*	0.04
Time 3 (5.5 years)	Group FR	1.02 (.38)*	2.78	1.38 (.46)*	3.96	–	–
	Language	−0.88 (.23)*	0.42	n/a	n/a	–	–
	Letter knowledge	n/a	n/a	−0.96 (.32)*	0.38	–	–
	Phonology	n/a	n/a	−0.72 (.32)*	0.49	–	–
	Constant	−2.07 (.33)*	0.13	−2.42 (.40)*	0.09	–	–
Time 4 (6–7 years)	Group FR	0.92 (.39)*	2.51	.887 (.48)	2.428	.91 (.51)**	2.5
	Language	−1.29 (.25)*	0.28	n/a	n/a	–	–
	Letter knowledge	n/a	n/a	−.856 (.35)*	0.425	−1.07 (.38)*	0.34
	Phonology	n/a	n/a	−1.050 (.32)*	0.35	−1.20 (.35)*	0.3
	RAN	n/a	n/a	−.770 (.29)*	0.463	−1.17 (.36)*	0.31
	Motor skills	n/a	n/a	n/a	n/a	−1.16 (.37)*	0.31
	Constant	−2.09 (.34)*	0.12	−2.398 (.41)*	0.091	−2.58 (.45)*	0.08

Model 1 was fitted using the Backward Wald procedure after entering risk group variable in the first step. Models 2 and 3 were fitted using the ENTER procedure after initially entering risk group variable in the first step. The abbreviation ‘n/a’ is used to indicate that the variable is not entered into the model. At T1 and T3, Model 2 was the best-fitting model and Model 3 is not shown.

* *p *=* *.05;

** *p *=* *.1.

Table3 (left-most columns) shows the classification outcomes according to the four age-specific language and FR models, using different cutoff values for the prediction probabilities. Following the procedure commonly adopted in screening studies (Puolakanaho et al., 2007), we display cutoff values of .50 and .25, and also present the cutoff that represents approximately 90% sensitivity (i.e., 90% of children with a reading disability were correctly identified). This is considered a reliable level for clinical purposes (Johnson, Jenkins, Petscher, & Catts, 2009). Model 1 (FR and language only as predictors) suffers a significant reduction in specificity at this level at each age (range 24.2–43.5%) indicating a high rate of false positives.

**Table 3:** Classification accuracy using different probability cutoffs for logistic models

Age phase	Model 1						Models 2/3					
	Probability level	Classification correct, %	Sensitivity, %	Specificity, %	True-Positive cases	False-positive cases	Probability level	Classification correct, %	Sensitivity, %	Specificity, %	True-Positive cases	False-positive cases
3.5	0.5	80.4	2.5	100	1	0	0.5	79.2	2.4	100	1	0
	0.25	68.3	57.5	71.1	23	46	0.25	66.7	61	68.2	25	48
	0.103	42.2	90	30.2	36	111	0.15	53.1	90.2	43	37	86
4.5	0.5	81.9	0	100	0	0	0.5	87.5	36.4	96.7	8	4
	0.25	74.4	47.2	80.4	17	32	0.25	86.1	68.2	89.3	15	13
	0.088	36.7	91.7	24.5	33	123	0.12	76.4	90.9	73.8	20	32
5.5	0.5	76.7	6.7	95.29	3	8	0.5	84.5	44.7	95.3	21	8
	0.25	74.9	62.22	78.2	28	37	0.25	79.5	63.8	83.7	30	28
	0.131	53.5	91.11	43.5	41	96	0.13	68.5	89.4	62.8	42	64
6–7	0.5	80.7	23.91	95.9	11	7	0.5	87.8	63.8	94	30	11
	0.25	77.1	69.6	79.1	32	36	0.25	84.3	76.6	86.3	36	25
	0.094	49.1	89.1	38.4	41	106	0.13	79	89.4	76.4	42	43

Probability cutoffs .25 and .5 are given as they are common in the literature. However, the best diagnostic models between T1 and T4 range from .08 to .15 cutoffs, giving 90% sensitivity in the sample.

Next, an ROC analysis was conducted (plotting sensitivity against false-positive rate to assess how well each age-specific version of Model 1 based over a range of cutoffs would perform. For 100% sensitivity and 100% specificity (a perfect screener), the area under the curve =1. Here, the values of the area under the curve from the ROC analysis were T1 – .689, T2 – .686, T3 – .749, and T4 – .789. Thus, these models are only moderately accurate in predicting dyslexia.

### Predicting dyslexia using familial risk and multiple predictors

The second set of models included the cognitive predictors of individual differences in reading. Once again, we fitted a series of logistic regression models using the same binary outcome, dyslexic/nondyslexic at T5. First, the core predictor variables are entered into the model along with family risk status: family risk in the first, RAN, phonology, and letter knowledge in the second block (Model 2; Table2, center columns). Second, a third block was added to investigate whether the inclusion of measures of executive or motor skills would improve the prediction of dyslexia (Model 3; Table2, right-most columns). Likelihood ratio (LR) tests were used to test for the additional benefit of the third block of predictors.

Table3 shows statistics for the age-specific logistic models which use the core predictors either alone (Model 2) or with executive and motor skills included (Model 3). The four models used to construct this table represent the best-fitting model at each time point (Model 2/Model 3) according to a likelihood ratio test (models with nonsignificant parameter estimates are not included). Significant predictors in the models were generally consistent with the findings of the *t*-tests. At age 3½ years (T1), only family risk status and letter knowledge were significant predictors (Model 2). At 4½ years (T2), the predictors producing the best-fitting model were family risk, phonology, letter knowledge, RAN, together with executive function skills (Model 3; change in LR test by 21.289 on 1 df). It is notable that with the addition of other predictors, the letter knowledge variable changes direction.

**Figure 2:**
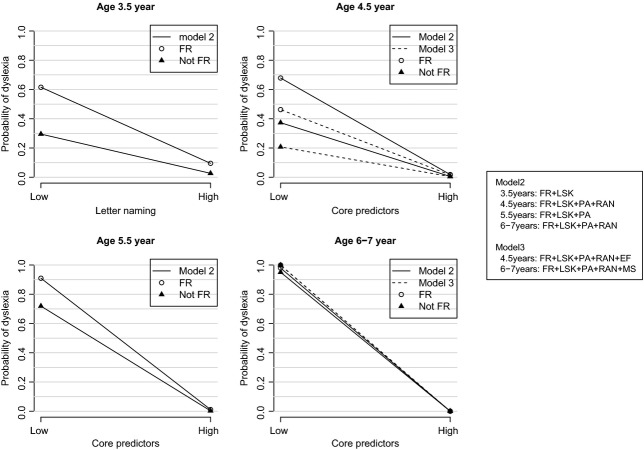
Age-specific probability curves showing the change in risk of dyslexia according to core predictors and family-risk status
*Notes*: Graphs represent changes in probability of identifying dyslexia at high and low levels of the predictor variable/s (*x-axis*) for the family risk (open circles) and not family risk (filled triangles) groups. Values represent high (+3) to low (−3) levels of performance. For the age-specific models at 3.5 years and 5.5 years, values are shown for Model 2 with (a) FR and letter knowledge; (b) FR, letter knowledge and phoneme awareness, respectively, as significant predictors. For the age-specific models at 4.5 years and 6–7 years, values are shown for both Model 2 (unbroken line) and Model 3 (dashed line). At age 4.5 years, Model 2 includes FR, letter knowledge, phoneme awareness, and RAN; Model 3 includes in addition, executive function measures. At age 6–7 years, Model 2 includes FR, letter knowledge, phoneme awareness, and RAN; Model 3 includes in addition, measures of motor skills.

At age 5½ (T3), the best-fitting model included family risk, phonology, and letter knowledge as predictors (RAN was not significant at this point when added into the model). At this time point, the addition of executive functions and motor skills (Model 3) did not produce a significantly better fit than Model 2. At age 6–7 years (T4), the three core predictors were again significant (Model 2) and adding motor skills as a predictor (Model 3) improved the model fit (change in 11.706 on 1 df). Importantly, at this time point, family risk status was not a significant predictor in the model. This is confirmed when examining the probability plots in Figure2. Only small differences are seen between the risk and no risk groups since the majority of differences are already explained by the other predictors.

The Nagelkerke *R*^2^ in the age-specific versions of Model 2 using family risk status and three core predictors ranged from 20% to 41% (T1 = 20.1%; T2 = 26.9%; T3 = 36.4%; T4 = 41.3%); however, versions of Model 3 – with the inclusion of executive skills at T2 and motor skills at T4 – led to an increase in *R*^2^ values (T2- 41.6%; T4 = 54.1%).

### Predicting individual risk of dyslexia

To examine how an individual’s risk of dyslexia changes according to the profile of skills and deficits that characterize their performance, we calculated the probabilities of dyslexia generated if high or low values (defined as +2 or −2 values on standardized composites of the core predictors, respectively) are chosen for the significant predictors and inserted into the formulae describing each logistic regression model. To highlight the differences between combinations of values for predictors, we entered either all high values or all low values (an individual with poor core skills would have low values) into the model together with family risk or no risk. This yielded four probabilities. These probabilities are plotted in Figure2 where, to aid comparison, we have joined corresponding points using different lines. The lines give a visual indication of how the probability of dyslexia differs when an individual has either high or low values on the predictor measures. It is clear from each plot that family-risk status plays an important role in predicting the probability of dyslexia up to T4, together with the core predictors (letter knowledge, phoneme awareness, and RAN) and executive skills at T2. However by T4, the core predictors plus motor skills alone provide accurate prediction.

**Figure 3:**
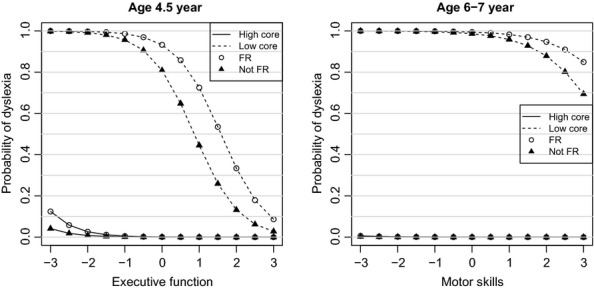
Age-specific probability curves showing the change in probability of identifying dyslexia when including measures of executive function (4½) and motor skills (6–7). High and low values of the core predictors and family risk status are used to emphasize their effects in the models
*Notes*: Graphs represent changes in probability of dyslexia at varying levels of the predictor variable/s (*x-axis*) for the family risk (open circles) and not family risk (filled triangles) groups according to whether children performed well on the core predictors (unbroken lines of lower curves) or poorly (dashed lines of upper curves).

Figure3 shows a more detailed probability curve that captures the effects of including executive function and motor skills as predictors at T2 and T4, respectively. Four specific cases are displayed in each plot as distinct curves; each curve represents combinations of family risk status or not, and high or low values of the core predictors. A very definite change is seen in both plots when the core predictors are fixed to low values. The most significant change in probability is observed when adding executive function at T2; here a strong sigmoidal curve is seen when core predictor values are low, but the change is less noticeable when core predictor values are high. At T4, the addition of motor skills has less effect on the change in probability over that based on the core predictors. The most significant effect can be seen at very low values of motor skills.

Finally, turning to the issue of sensitivity and specificity, Table3 (right hand columns) shows the classification of outcomes according to the four best fitting age-specific models that include the core and additional predictors (removing language). As stated earlier, the cutoff values, .50, .25, and the cutoff that represents 90% sensitivity are presented for the predicted probabilities of having dyslexia. An improvement in classification has been achieved over the simple model including just family risk and language yielding much higher and clinically reliable values for specificity (range 43% at 3½ to 76.4% at 6–7). The corresponding ROC analysis, assessing how well each model performed across a range of cutoffs, produced values for area under the curve of: T1- .707, T2-.875, T3-.850, and T4- .910.

## Discussion

In this study, we followed the development of children at high risk of dyslexia and classified them according to literacy outcome using a cutoff of 1.5SD below that of children in a low-risk control group. We hypothesized that children at family risk of dyslexia and those with language difficulties would have a high probability of developing reading problems. In addition, those who performed poorly with respect to three skills known to be proximal causes of poor reading, namely, letter knowledge, phonological awareness, and rapid naming would likely be affected. We sought evidence of which tasks at different ages produced the best prediction of dyslexia and whether deficits in executive or motor skills, commonly comorbid with language impairment, would further increase the likelihood of dyslexia.

The children in our sample at high risk of dyslexia were drawn from three groups: children at family risk of dyslexia, children with poor language in preschool and children with both risk factors. When information regarding family risk and language skills was all that was available to predict later dyslexia in a sample also containing ‘not at risk’ children, we found that family-risk status (as a dichotomous variable) was predictive of dyslexia outcome at every time point, whereas language skill (a continuous measure) was not a significant predictor until age 5 years. These findings suggest that language difficulties can be considered a significant risk factor for dyslexia around the time of school entry, but do not discriminate well between those who will and will not develop dyslexia at an earlier stage of development. Thus, a preschool screening measure for dyslexia including only family-risk status and language skills would be prone to over identify those likely to go on to have reading problems. Indeed, consistent with Snowling, Gallagher, and Frith (2003), the model containing only language measures and FR status suffers a significant reduction in specificity at 90% sensitivity and therefore is less than adequate for the purposes of early identification.

The models which included known proximal predictors of reading and dyslexia provided better prediction, consistent with Puolakanaho et al. (2007) who reported parameter estimates comparable in scale and magnitude and Pennington and Lefly (2001) who showed that a two-step procedure, first ascertaining family risk of dyslexia and second, introducing predictors of reading (letter knowledge, phonological awareness, RAN, speech perception, and verbal short-term memory) at 5 years yielded 74% sensitivity and 87% specificity. As in the Finnish sample of children at family risk of dyslexia studied by Puolakanaho et al. (2007), the best-fitting model differed for different ages of assessment. At age 3½ years (T1), we found that a model containing only family-risk status and letter knowledge best predicted dyslexia outcome. At older ages, measures of phonological awareness and sometimes RAN increased the probability of accurate identification.

Our findings extend the work of Puolakanaho et al. (2007) in two ways. First, over and above the established predictors of reading, we found that executive skills at age 4½ improved the prediction of dyslexia in the current sample; moreover, when compared with the best-fitting model at the same age reported by Puolakanaho et al. (2007), it produced higher specificity (73.8% compared with 56.6%) at 90% sensitivity. Second, and importantly, at age 6, family risk status was no longer a significant predictor of dyslexia when core cognitive skills were in the model, but motor skills added to the probability of correct prediction, perhaps through its impact on writing and hence spelling. Thus, the best-fitting age-specific models producing adequate sensitivity and specificity used measures at ages 4½, and 6–7 years. Both models included the core cognitive predictors of reading (letter knowledge, phoneme awareness and RAN): at 4½ years, the model also including executive skills and family risk status and yielded 91% sensitivity, 73% specificity, and 76.4% classification. At 6–7 years, the core predictors with motor skills (and not family risk) produced somewhat better classification of 79% with 89% sensitivity, 76% specificity.

The possible reasons why executive and motor skills only contributed to the best-fitting models at a single time point each requires further investigation. We speculate that although executive skills were only predictors over and above core reading-related skills at 4½ years, it is likely that self-regulation and attentive skills predict children’s readiness for formal schooling at this age. Indeed, the effects of individual differences in executive skills were particularly clear when a child did poorly on the core predictors of dyslexia, suggesting good executive control provides some compensation for the impact of low ‘readiness’ for learning to read. Similarly, it seems likely that motor skills involved in developing good pencil control may facilitate the development of early literacy skills at around 6 years when they are in ascendancy, but be less relevant at earlier developmental stages.

The study had a number of limitations. First, the data are drawn from a high-risk sample and therefore it is unclear how they would generalize to a population sample. Second, methodological differences between studies also preclude comparison of some of the findings. For example, our finding that family-risk status was not a good predictor of dyslexia outcome over and above cognitive measures seems at odds with other studies in the literature. There are several possible explanations for this. First, we are predicting dyslexia outcome as a category rather than a dimension (cf. Carroll et al., 2014); second, we are predicting from models at different ages and containing different predictors (cf. Pennington & Lefly, 2001; Puolakanaho et al., 2007). Nonetheless, the findings highlight what makes children vulnerable to early difficulties in learning to decode and to spell (the cardinal features of dyslexia) and highlights factors clinicians can take into account when trying to identify children at risk of reading difficulties. These include a family history of dyslexia, delayed language development and difficulties in learning letters and in reflecting on the sound structure of spoken words.

Together our findings have important implications for public health. The first concerns the value of screening for dyslexia in the preschool years. It is clear from our analyses that knowing whether or not a child is at family risk of reading problems can provide an indication from a relatively young age – here around 3½ years – that they are likely to experience difficulties learning to read. If they also have poor knowledge of letters this is a second indicator of such risk. While reference to these two pieces of information is likely to over identify dyslexia, the cost of screening is low. In the current project, we have demonstrated that a home literacy environment rich in books and print-related interactions is associated with a good start in word decoding and comprehension (e.g., Hamilton, 2013). It follows that positive screening could lead to advice as to how best parents can support their child’s emergent reading skills. Equally it is important that once risks are identified, progress is monitored in the early stages of formal reading instruction to insure that letter knowledge and phoneme awareness skills are acquired and if not, to provide specialist teaching to overcome any difficulties with these foundational skills (Snowling & Hulme, 2011; for a review). On the other hand, our findings suggest that screening for language problems at 3½ years provides less useful information at least *with respect to later dyslexia*. Given that many children with language impairments experience reading problems, this finding may appear counter-intuitive. However, it needs to be borne in mind that language delays and difficulties may resolve by school entry (e.g., Bishop & Edmundson, 1987). Consistent with this, we found language to be an important predictor of dyslexia when measured at ages 5½ through 7 years but not before. A second important message of this study for public health is that children who show persistent problems with speech and language at school entry are at high risk of difficulties with literacy and these require systematic intervention (Rose, 2009). More generally, language and communication are critical foundations for many aspects of school adjustment as well as being predictors of arithmetic and reading comprehension skills. Our current findings should therefore not be taken to argue against preschool screening for language learning impairments which is of prime importance.

## Conclusion

Our findings highlight the fact that family risk of dyslexia is a strong predictor of reading outcome. Equally, it is clear that early identification of ‘dyslexia’ is difficult and the closer assessments are to school entry the more accurate predictions become. An important finding is that early language delay, an established risk factor for dyslexia, is not a good predictor at the individual level until close to school entry. This evidence is consistent with the view that many children with delayed language who resolve their difficulties learn to read normally though it is important to remember that such children remain at risk of poor reading comprehension and a wide range of other difficulties, including social problems.

**Key points**

- Family history of dyslexia is a predictor of literacy outcome from the preschool years.
- Children with language difficulties which persist at school entry are at high risk of dyslexia.
- Letter knowledge, phonological awareness and rapid naming skill provide a good screening battery in early primary school.
- Dyslexia is the outcome of multiple risk factors. Good executive and motor skills can be protective for children who have weaknesses in letter knowledge, phonological awareness, or rapid naming skills.

## Supporting Information

**Appendix S1.** Participant flow. **Table S1.** Means, standard deviation and range for measures across the whole sample.
