# Supplementary material for: Developmental dyslexia: predicting individual risk
Source: J Child Psychol Psychiatry. 2015 Apr 2;56(9):976–87. doi: 10.1111/jcpp.12412 (PMC4672694; doi:10.1111/jcpp.12412)
Supplement: Supplementary file 1 [file jcpp0056-0976-sd1.docx]

**Online supplementary material for “Developmental dyslexia: predicting individual risk” / Thompson et al.**

[**Appendix S1 Participant Flow**](#App_S1)
[**Table S1 Means, standard deviation and range for measures across the whole sample**](#Table_S1)

**Appendix S1**Participant Flow

**TD referral group**

No history of dyslexia

No current speech/language concerns

N = 76

**FR referral group**

Family history of dyslexia

Took precedence over parental concerns re speech/language (N=53)

N = 123

**SLI referral group**

Parental report of speech/language concerns

N = 46

**Control**

**N = 71**

**FRSLI**

**N = 37**

**FR**

**N = 86**

**SLI (not)**

**N = 15**

**SLI**

**N = 36**

**Research criteria for SLI**

T1 referral

**Withdrawals at T2 N = 16**

**2 control, 3 FR, 7 FRSLI, 4 SLI**

**Enter at T2**

**5 control , 7 FR, 1 FRSLI, 1 SLI, 1 SLI (not)**

T1 data

N = 245

T2 data

N = 244

**SLI**

**N = 33**

**Control**

**N = 74**

**FRSLI**

**N = 31**

**FR**

**N = 88**

**SLI**

**N = 33**

**SLI (not)**

**N = 15**

**Withdrawals at T3 N = 3**

**2 FR, 1 SLI (not)**

**Withdrawals at T4 N = 2**

**1 FR, 1 SLI**

**Control**

**N = 74**

**FRSLI**

**N = 31**

**FR**

**N = 87**

**SLI**

**N = 32**

**SLI (not)**

**N = 15**

T3 data

N = 241

T4 data

N = 239

**Control**

**N = 74**

**FRSLI**

**N = 31**

**FR**

**N = 90**

**SLI (not)**

**N = 16**

**FRSLI**

**N = 30**

**FR**

**N = 86**

**SLI**

**N = 30**

**SLI (not)**

**N = 15**

T5 data

N = 234

**Control**

**N = 73**

**Withdrawals at T5 N = 5**

**1 control, 1 FR, 1 FRSLI, 2 SLI**

**Table S1** *Means, standard deviation and range for measures across the whole sample*

|  | N | Mean | SD | Min | | Max |  |  |
| --- | --- | --- | --- | --- | --- | --- | --- | --- |
| *time 1* |  |  |  |  | |  |  |  |
| WPPSI block design^1^ | 227 | 103.81 | 19.26 | 58 | | 154 |  |  |
| WPPSI object assembly^1^ | 229 | 110.14 | 16.42 | 70 | | 154 |  |  |
| CELF basic concepts^1^ | 227 | 99.97 | 19.19 | 46 | | 124 |  |  |
| CELF sentence structure^1^ | 226 | 96.68 | 17.62 | 46 | | 136 |  |  |
| CELF expressive vocabulary^1^ | 225 | 100.77 | 19.11 | 58 | | 142 |  |  |
| PSrep std score ^1^ | 213 | 88.02 | 20.00 | 49 | | 130 |  |  |
| Letter Knowledge ^2^ | 230 | 2.88 | 3.47 | 0 | | 12 |  |  |
| visual search efficiency | 218 | 0.10 | 0.07 | -.12 | | .25 |  |  |
| GONOGO efficiency | 173 | 0.45 | 0.09 | .06 | | .50 |  |  |
| HTKS complex inhibition ^3^ | 169 | 9.06 | 10.96 | 0 | | 40 |  |  |
| copying shapes^4^ | 228 | 1.44 | 1.04 | 0 | | 3 |  |  |
| ABC coin^5^ | 229 | 31.84 | 9.34 | 17 | | 60.57 |  |  |
| ABC beads^5^ | 221 | 62.39 | 26.11 | 23 | | 127.02 |  |  |
| ABC bike trails | 220 | 9.40 | 4.62 | 0 | | 19.11 |  |  |
| ABC coin nonprefer^5^ | 220 | 35.72 | 9.65 | 18 | | 59.31 |  |  |
| *time 2* |  |  |  |  | |  |  |  |
| ROWPVT^1^ | 228 | 107.29 | 11.94 | 66 | | 134 |  |  |
| CELF sentence structure^1^ | 229 | 103.54 | 16.44 | 46 | | 148 |  |  |
| ESIT^6^ | 209 | 5.72 | 4.02 | 0 | | 19 |  |  |
| PI phoneme isolation ^7^ | 180 | 7.62 | 5.44 | 0 | | 16 |  |  |
| AM alliteration matching ^8^ | 219 | 7.26 | 2.19 | 1 | | 10 |  |  |
| RAN objects rate^9^ | 207 | 0.69 | 0.19 |  | | 1 |  |  |
| RAN colours rate^9^ | 191 | 0.60 | 0.19 |  | | 1 |  |  |
| Letter knowledge ^1^ | 227 | 108.15 | 15.33 | 72 | | 132 |  |  |
| Block recall^5^ | 220 | 15.68 | 3.96 | 1 | | 24 |  |  |
| visual search efficiency | 223 | 0.16 | 0.07 | -.08 | | .30 |  |  |
| ACPT efficiency | 213 | 0.14 | 0.11 | -.48 | | .25 |  |  |
| HTKS^10^ | 218 | 20.28 | 12.29 | 0 | | 39 |  |  |
| ABC beads^5^ | 226 | 40.21 | 14.96 | 13 | | 80.33 |  |  |
| ABC bike trails | 227 | 4.79 | 3.74 | 0 | | 13.49 |  |  |
| ABC coin^5^ | 228 | 24.57 | 5.16 | 14 | | 39.88 |  |  |
| ABC coin nonprefer^5^ | 228 | 28.03 | 6.70 | 17 | | 46.43 |  |  |
| *time 3* |  |  |  |  | |  |  |  |
| CELF expressive vocabulary^1^ | 226 | 108.63 | 17.53 | 52 | | 148 |  |  |
| CELF sentence structure^1^ | 226 | 105.92 | 17.22 | 64 | | 142 |  |  |
| ESIT^6^ | 222 | 8.17 | 4.71 | 0 | | 19 |  |  |
| phoneme isolation^7^ | 226 | 12.56 | 4.31 | 0 | | 16 |  |  |
| YARC phoneme deletion ^11^ | 226 | 6.48 | 2.75 | 0 | | 12 |  |  |
| Letter Knowledge^1^ | 226 | 110.31 | 13.95 | 68 | | 129 |  |  |
| RAN objects rate^9^ | 222 | 0.98 | 0.26 |  | | 2 |  |  |
| Block recall^5^ | 226 | 19.00 | 3.86 | 9 | | 30 |  |  |
| visual search efficiency | 223 | 0.20 | 0.06 | -.10 | | .30 |  |  |
| ACPT efficiency | 217 | 0.10 | 0.07 | -.24 | | .17 |  |  |
| HTKS^10^ | 223 | 27.93 | 10.12 | 0 | | 39 |  |  |
| ABC beads^5^ | 226 | 32.90 | 9.90 | 16 | | 55.68 |  |  |
| ABC bike trails^5^ | 225 | 5.11 | 4.23 | 0 | | 15.32 |  |  |
| ABC coin | | 226 | 24.49 | 4.86 | | 15 | 36.68 | |
| ABC coin nonprefer^5^ | | 225 | 26.85 | 5.38 | | 17 | 41.44 | |
| *time 4* | |  |  |  | |  |  | |
| ROWPVT^1^ | | 226 | 108.66 | 13.49 | | 69 | 146 | |
| CELF expressive vocabulary^1^ | | 226 | 104.38 | 17.33 | | 46 | 148 | |
| CELF word structure^1^ | | 226 | 106.37 | 16.46 | | 46 | 130 | |
| ESIT^6^ | | 226 | 9.92 | 4.85 | | 0 | 20 | |
| TROG^1^ | | 226 | 95.85 | 20.04 | | 55 | 134 | |
| YARC phoneme deletion^12^ | | 226 | 8.07 | 2.74 | | 0 | 12 | |
| RAN objects rate^9^ | | 224 | 0.95 | 0.22 | |  | 2 | |
| RAN digits rate^9^ | | 223 | 1.30 | 0.39 | |  | 3 | |
| Letter knowledge^1^ | | 226 | 110.88 | 12.79 | | 68 | 124 | |
| Block recall^5^ | | 226 | 20.51 | 4.25 | | 6 | 30 | |
| Visual search efficiency | | 225 | 0.26 | 0.08 | | -.10 | .47 | |
| ABC coin^5^ | | 226 | 21.16 | 4.47 | | 12 | 38.20 | |
| ABC coin nonprefer^5^ | | 226 | 22.65 | 4.67 | | 11 | 35.05 | |
| ABC beads^5^ | | 226 | 26.28 | 7.63 | | 13 | 43.68 | |

*Notes*: 1. standard score; 2 max = 12; 3 max = 40;4 max = 3; 5 = raw score; 6 max= 30; 7 max =16; 8 max = 10; 9 items/sec; 10 max = 40; 11 max = 12; 12 max = 12.
